# Interaction of *Rhus typhina* Tannin with Lipid Nanoparticles: Implication for the Formulation of a Tannin–Liposome Hybrid Biomaterial with Antibacterial Activity

**DOI:** 10.3390/jfb14060296

**Published:** 2023-05-25

**Authors:** Szymon Sekowski, Nikolaos Naziris, Maria Chountoulesi, Ewa Olchowik-Grabarek, Krzysztof Czerkas, Artem Veiko, Nodira Abdulladjanova, Costas Demetzos, Maria Zamaraeva

**Affiliations:** 1 Laboratory of Molecular Biophysics, Department of Microbiology and Biotechnology, Faculty of Biology, University of Bialystok, 15-245 Bialystok, Poland; ewaolch@uwb.edu.pl (E.O.-G.); kc1802@wp.pl (K.C.);; 2 Section of Pharmaceutical Technology, Department of Pharmacy, School of Health Sciences, National and Kapodistrian University of Athens, Panepistimioupolis Zografou, 15771 Athens, Greece; niknaz@pharm.uoa.gr (N.N.); mchountoules@pharm.uoa.gr (M.C.); demetzos@pharm.uoa.gr (C.D.); 3 Department of General Biophysics, Faculty of Biology and Environmental Protection, University of Lodz, Pomorska 141/143, 90-236 Lodz, Poland; 4 Department of Biochemistry, Yanka Kupala State University of Grodno, Bulvar Leninskogo Komsomola, 5, 230030 Grodno, Belarus; veiko_ag@grsu.by; 5 Institute of Bioorganic Chemistry, Academy of Sciences of the Republic of Uzbekistan, Tashkent 100143, Uzbekistan; anodira73@rambler.ru

**Keywords:** liposomes, sumac tannin, biomaterials, hybrid nanosystems, antibacterial activity

## Abstract

Tannins are natural plant origin polyphenols that are promising compounds for pharmacological applications due to their strong and different biological activities, including antibacterial activity. Our previous studies demonstrated that sumac tannin, i.e., 3,6-bis-O-di-O-galloyl-1,2,4-tri-O-galloyl-β-D-glucose (isolated from *Rhus typhina* L.), possesses strong antibacterial activity against different bacterial strains. One of the crucial factors of the pharmacological activity of tannins is their ability to interact with biomembranes, which may result in the penetration of these compounds into cells or the realization of their activity on the surface. The aim of the current work was to study the interactions of sumac tannin with liposomes as a simple model of the cellular membrane, which is widely used in studies focused on the explanation of the physicochemical nature of molecule–membrane interactions. Additionally, these lipid nanovesicles are very often investigated as nanocarriers for different types of biologically active molecules, such as antibiotics. In the frame of our study, using differential scanning calorimetry, zeta-potential, and fluorescence analysis, we have shown that 3,6-bis-O-di-O-galloyl-1,2,4-tri-O-galloyl-β-D-glucose interacts strongly with liposomes and can be encapsulated inside them. A formulated sumac–liposome hybrid nanocomplex demonstrated much stronger antibacterial activity in comparison with pure tannin. Overall, by using the high affinity of sumac tannin to liposomes, new, functional nanobiomaterials with strong antibacterial activity against Gram-positive strains, such as *S. aureus*, *S. epidermitis,* and *B. cereus*, can be formulated.

## 1. Introduction

Tannins are secondary plant metabolites which are, structurally, a diverse group of polyphenols represented by hydrolysable tannins, condensed tannins, phlorotannins, complex tannins [1], and by gallocatechins and their gallates [2]. The scientific interest in this group of plant phytochemicals has recently increased, owing to their various beneficial effects on health, such as antitumorigenic, antioxidative, anticlotting, anti-inflammatory, antiviral, and antimicrobial effects [1,3,4,5].

One promising compound in this category, in terms of pharmacological properties, is the 3,6-bis-O-di-O-galloyl-1,2,4-tri-O-galloyl-β-D-glucose—hydrolysable sumac tannin, which is isolated from *Rhus typhina* (staghorn sumac) leaves, a plant widely used in traditional and veterinary medicine, especially in Asia [6]. Sumac tannin is structurally similar to the well-studied 1,2,3,4,5-penta-O-galloyl-β-d-glucose (PGG). However, in contrast to PGG, it contains 3 gallic acid and 2 digallic acid residues linked to glucose (19 and 15-OH groups, respectively) compared to the 5 gallic acid residues present in the PGG structure.

Our previous studies have shown that 3,6-bis-O-di-O-galloyl-1,2,4-tri-O-galloyl-β-D-glucose, herein after referred as hydrolysable *Rhus typhina* tannin (RT), exhibits antimicrobial activity and prevents hemolysis caused by bacterial toxins [7,8] and osmotic shock [9]. RT has also shown high and specific antiradical activity relative to reactive oxygen species (ROS) and reactive nitrogen species (RNS), as well as a protective effect on red blood cells against oxidative stress caused by a variety of oxidants, including bisphenol A (BPA) and its metabolite hydroquinone [10,11].

We have also demonstrated that RT, due to its ability to bind to proteins and in particular to α-synuclein protein, prevents the protein aggregation, exhibiting potential neuroprotective activity in Parkinson’s disease [12] and preventing albumin against glycation and protecting Neuro2A nerve cells against oxidative stress induced by high glucose levels [13]. It has been also proven that a water-acetonic extract from *Rhus typhina* leaves containing more than 70% RT exhibited low toxicity levels (LD_50_ = 5600 mg/kg) and an antitumor effect [14]. The pharmacological activity of polyphenols is associated with their ability to interact with biomembranes, which may result in either penetration of the compound in the cells or action on cells’ surfaces. In both cases, polyphenol–membrane interaction leads to a change in the physicochemical properties of the membrane and in their functionality in total. Lipids play a significant role in the interaction of polyphenols with membranes, as they compose the main component of the membrane framework and are responsible for properties such as fluidity, phase transition temperature, stability, surface, and the dipole potential of the membrane. A plethora of studies have shown that a polyphenol-induced decrease in membrane fluidity inhibited the distribution of free radical reaction of fatty acids oxidation and leads to a cessation of oxidative stress [15,16,17,18,19]. Condensed tannins have been shown to exert an antidifferentiation effect on preadipocytes by disruption of the membrane integrity and an increase in membrane fluidity [20]. Several research groups have documented that the cytotoxic activity of polyphenols with a number of cancer cells is related to their lipophilicity and affinity for lipids [21]. A correlation has been shown between the antibacterial activity of catechin derivatives and their ability to affect the physical properties of the phospholipid membrane [22,23]. In our previous investigation, we have proven the relation between the antihemolytic activity of RT against *Staphylococcus aureus* cytolysins and the stiffening of the hydrophobic part of the erythrocyte membrane [8].

Liposomes are a widely accepted model for biological membranes and a convenient test system for studying the activity of compounds, which are considered to act through modification of the physicochemical properties of the membranes [24]. It must be emphasized that studying the interactions of active compounds (e.g., drugs or polyphenols) with membranes is fundamental for assessing their localization in the membranes and their effects on the membrane structure and surface potential, which are critical parameters in the potential pharmacological implementation of polyphenols. In addition, liposomes are very often investigated as nanocarriers for different types of biologically active molecules, such as antibiotics, and can contribute to an increase in the absorption of the drug. A significant increase in bacterial resistance to antibiotics leads to the constant search for new antimicrobial agents among compounds of plant origin, including polyphenols along with their new formulations and modifications. 

The aim of this work was to investigate the interaction of RT with artificial lipid nanovesicles (liposomes) prepared from dimyristoylphosphatidylcholine (DMPC), which is present in mammalian membranes and is, thus, used as a simple model of cell membranes [25] to determine the influence of RT on their biophysical parameters, as well as to study the antibacterial activity of a newly formulated, hybrid RT–liposome nanobiomaterial.

## 2. Materials and Methods

### 2.1. Materials

**Figure 1:**
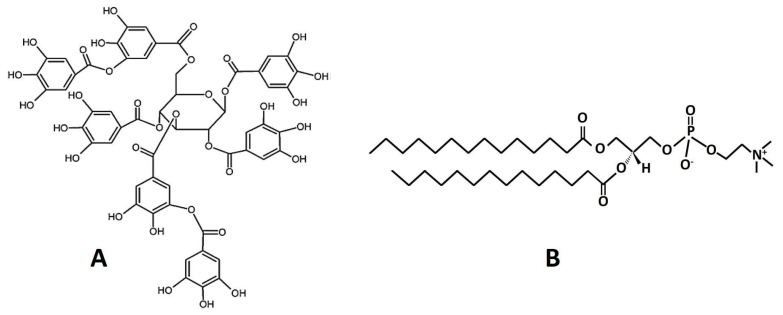
(**A**) Molecular structure of 3,6-bis-O-di-O-galloyl-1,2,4-tri-O-galloyl-β-D-glucose (RT), (**B**) chemical structure of 1,2-dimyristoyl-sn-glycero-3-phosphocholine (DMPC).

DMPC (1,2-dimyristoyl-sn-glycero-3-phosphocholine) was purchased from Avanti Polar Lipids (Alabaster, AL, USA). A noncommercial sumac tannin (3,6-bis-O-di-O-galloyl-1,2,4-tri-O-galloyl-β-D-glucose) (Figure 1) was isolated from *Rhus typhina,* according to the method proposed by Olchowik-Grabarek et al. [8]. TMA-DPH (1-(4-trimethylammoniumphenyl)-6-phenyl-1,3,5-hexatriene), DPH (1,6-diphenyl-1,3,5-hexatriene), and Laurdan were purchased from Sigma-Aldrich (St. Louis, MO, USA). All other compounds were of analytical grade.

### 2.2. Liposome Preparation

Liposomes were prepared in two different ways, depending on the analysis to be performed. For electrophoretic and dynamic light scattering (ELS and DLS) studies, liposomal formulations of DMPC and DMPC:RT in various molar ratios were prepared by utilizing the thin-film hydration and sonication method. Briefly, DMPC in chloroform and the 3,6-bis-O-di-O-galloyl-1,2,4-tri-O-galloyl-β-D-glucose in methanol were mixed and then transferred into a round flask and connected to a rotary evaporator (Rotavapor R-114, Buchi, Flawil, Switzerland). A vacuum of −1 bar was applied and the thin film was formed with slow removal of the solvent at 40 °C. The film was maintained under vacuum for at least 30 min to remove traces of solvent and stored at 4 °C overnight. Subsequently, it was hydrated with PBS (pH = 7.4) by slowly stirring for 1 h in a water bath, above the phase transition temperature of the lipid (24 °C for DMPC), with a lipid concentration of 30 mg/mL. The resultant structures (apparently multilamellar vesicles, MLVs) were subjected to 2, 5 min sonication cycles (amplitude 70%, cycle 0.5 s) interrupted by a 5 min resting period, using a probe sonicator (UP 200S, Dr. Hielsher GmbH, Berlin, Germany). The resultant particles (tentatively assigned as small unilamellar vesicles, SUVs) were allowed to anneal for 30 min. The prepared systems of lipids and tannins were assigned as DMPC:RT1, DMPC:RT5 and DMPC:RT10.

For fluorescence studies of the interaction between RT and DMPC and for antibacterial analysis, liposomes were prepared using the extrusion method by using the Avanti Polar Lipids Mini-Extruder, according to Sekowski et al. [26]. Generally, DMPC phospholipids were dissolved in chloroform and then the solvent was evaporated. The formed thin lipid film was purged by nitrogen, resuspended in PBS, well mixed, and afterward heated to 45 °C, passing 15 times through the extruder polycarbonate membrane (pore diameter 100 nm). The final DMPC concentration was 20 mg/mL. Liposomes were stored at 4 °C and, for the experiments, were diluted up to 100 µg/mL. For the antibacterial studies. the liposomes were prepared in practically the same manner, with small modifications, i.e., the pure PBS used RT solution in PBS (C = 2 mM) and was added to the lipid film. After mixing well, the lipid–RT solution was heated up to 40 °C and then passed 15 times through an extruder (Avanti Polar Lipids) that possessed 100 nm pore diameter membranes. The final concentrations of DMPC and RT in the formulated nanosystem were 20 mg/mL and 2 mM, respectively.

### 2.3. Analysis of ζ-Potential and Particle Size—Light Scattering

The size, size distribution, and ζ-potential of the obtained structures were investigated using dynamic and electrophoretic light scattering (DLS and ELS). The physicochemical parameters were measured immediately after preparation (t = 0 days), as well as over time (t = 5 days), for the monitoring of the system’s physical stability. For DLS and ELS, 100 μL or 50 μL aliquots were 30-fold and 60-fold diluted in HPLC-grade water, respectively. Measurements were performed at 25 °C at a detection angle of 90° using a photon correlation spectrometer (Zetasizer 3000 HSA, Malvern, UK) and were analysed with the CONTIN method (MALVERN Pananalytical Ltd., software). Details on the methods have been previously published [27].

### 2.4. Preparation of Lipid Bilayers

Pure lipid DMPC and mixed DMPC:RT bilayers were prepared by mixing the appropriate amounts of DMPC and tannin. DMPC bilayers were DSC-analysed as they were but also after hydration with tannin PBS solution in different concentrations (described below). Specifically, DMPC bilayers were prepared with the evaporation of the DMPC solution in chloroform (10 mg/mL) at 60 °C and were further dried at 40 °C for 30 min. A mixed DMPC:RT bilayer was prepared by adding the appropriate amount of tannin in methanol to the DMPC solution in chloroform and followed the same evaporation method. This sample was designed to include the tannin incorporated inside the DMPC bilayers. The obtained laminated bilayers were hydrated into the appropriate aqueous medium (PBS) and then studied using differential scanning calorimetry (DSC).

### 2.5. Differential Scanning Calorimetry (DSC)

DSC thermograms of DPMC bilayers, neat, with incorporated tannin or with post-evaporation-added tannin were obtained by utilizing a DSC822^e^ Mettler Toledo (Schwerzenbach, Switzerland) calorimeter, calibrated with pure indium (T_m_ = 156.6 °C). A Sealed 40 μL aluminum crucibles were used as sample holders. The systems under investigation were bilayers composed of DMPC and the tannin molecule in various molar concentrations, i.e., to get 1 µM; 5 µM and 10 µM of RT per 100 µg/mL of lipids. Initially, around 3 mg of dried samples was weighted and placed in a crucible, followed by hydration with, accordingly, 30 μL of PBS, with or without dissolved tannin and sealing of the crucible. Then each prepared sample was left to equilibrate for a 15 min period prior to measurement. In the case of the incorporated tannin, the dried sample already included the molecule and was hydrated with PBS. The reference for the measurement of every sample was an empty aluminium crucible. Two heating–cooling cycles were performed, and reproducibility of the sample analyses was achieved. The temperature range used was from 5 °C to 35 °C and the scanning rate was 2.5 °C/min. Before each cycle, the samples were subjected to a constant temperature of 5 °C for 5 min. to ensure equilibration. The calorimetric data obtained (characteristic transition temperatures T_onset,m/s_ and T_m/s_, enthalpy changes ΔH_m/s_, and widths at half peak height of the C_p_ profiles ΔT_1/2,m/s_) were analysed using Mettler Toledo STAR^e^ software. It is noted that the transition enthalpy is expressed as kilojoules per moles of DMPC and is considered positive during an endothermic process.

### 2.6. RT–DMPC Interaction Studies: Fluorescence Analysis of Lipid Order, Nanodomain Formation, and Biophysical Parameters

Lipid order parameters were defined by the measurement of fluorescence anisotropy ® changes of TMA-DPH and DPH. Liposomes (C = 100 µg/mL) were incubated for 20 min with 1 µM of TMA-DPH (dissolved in methanol) or DPH (dissolved in tetrahydrofuran) and the fluorescence anisotropy for pure liposomes and, after the addition of RT (in the concentration range 0.5–10 µM), was measured. Fluorescence excitation and emission wavelengths were λ_exc._ = 340 nm, λ_em._ = 430 nm (for TMA-DPH), and λ_exc._ = 348 nm, λ_em._ = 426 nm (for DPH). Based on the “r” values, the lipid order parameter (S) was calculated as we described previously [8].

Nanodomain formation was studied using fluorescence staining by Laurdan. Briefly, DMPC liposomes (C = 100 µg/mL) were stained by Laurdan (at final concentration of 400 nM), mixed, and incubated for 5 min. Next, the fluorescence signal from the pure liposomes and liposomes in the presence of sumac tannin was analysed using λ_exc._ = 350 nm and 2 emission wavelengths: ^1^ λ_em._ = 440 nm and ^2^ λ_em._ = 490 nm.

Biophysical parameters characterizing DMPC–RT interactions were calculated based on fluorescence quenching of TMA-DPH. Liposomes at final concentration of C = 100 µg/mL were labelled using TMA-DPH at final concentration of 400 nM and incubated for 20 min at 25 °C. After incubation, fluorescence of liposomes without and in the presence of sumac tannin was measured by using λ_exc._ = 340 nm and λ_em._ = 430 nm.

### 2.7. Antibacterial Activity of RT–DMPC Hybrid Nanosystems

As controls, pure DMPC liposomes and pure RT (both in PBS) were used. The antimicrobial activity was examined on the six bacteria strains: *Staphylococcus aureus* ATCC 700699; *Staphylococcus aureus* 8325-4; *Staphylococcus epidermitis* ATCC 14990; *Bacillus cereus* ATCC 13061; *Escherichia coli* ATCC 35218; and *Pseudomonas aeruginosa* ATCC BAA-1744. Analyses were performed according to Czajkowska-Szczykowska et al. [28]. Briefly, the compounds (RT–DMPC nanocomplex and pure RT as control) were added to a Mueller Hinton broth (MHB) medium for the bacteria to a final RT concentration of 500 µM and 5 mg/mL (7.4 mM) of DMPC. The samples were then serially 2-fold diluted (12-times) in 96-well microtiter plates with final volumes of 100 μL. Next, 100 μL of bacteria solution was added to get the final bacteria cell concentration at 1 × 10^6^ colony-forming units per mL (CFU/mL). The plates were incubated at 37 °C for 24 h. The minimum inhibitory concentration (MIC) value was determined as the lowest concentration of an antibacterial agent that inhibited bacterial growth, as indicated by the absence of turbidity.

## 3. Results and Discussion

### 3.1. Physicochemical and Thermodynamic Characterization of Liposomes That Contain RT

It is widely known that polyphenols interact strongly with both the cells and model membranes [8,26,29,30,31,32]. These interactions lead to changes in membrane physicochemical parameters such as fluidity, surface charge, transition temperature, and lipid order parameters [8,26].

We have previously shown that RT exhibits a wide range of biological activity on different type of cells, including antibacterial and antiglycation [8,13].

Therefore, the interaction of RT with DMPC nanovesicles, which are used as a simple model of cell membranes due to their high abundance in mammalian membranes [25], has been studied to better investigate and understand the physicochemical nature of this activity.

In order to verify if RT can influence the electrical properties of lipid membranes, their size, size distribution, and ζ-potential were measured using electrophoretic and dynamic light scattering (ELS and DLS). Measurements were performed immediately after preparation (0 days) as well as after 5 days to check the physical stability of the DMPC liposome–RT mixture.

**Figure 2:**
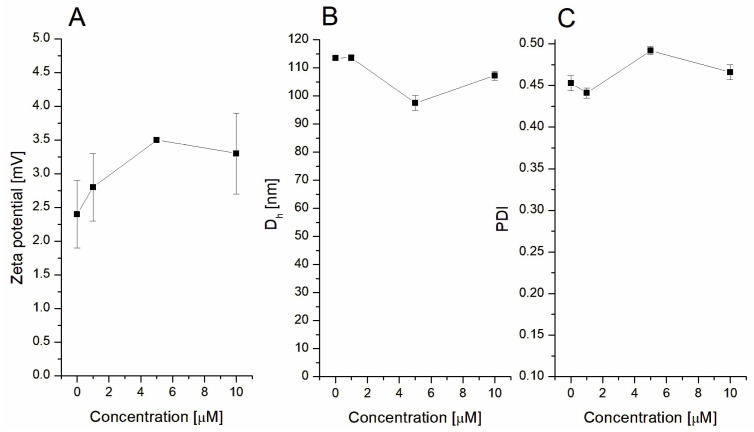
Zeta-potential changes of pure DMPC and DMPC–RT mix (**A**), hydrodynamic diameter (**B**), and polydispersity index (PDI) (**C**). The *X*-axis demonstrates the final RT concentration.

The day of the liposome preparation (day 0), both the pure DMPC-vesicles and DMPC–RT mixtures formed homogenous, almost transparent colloidal suspensions. For the pure DMPC liposomes, analysis of ζ-potential demonstrated an almost neutral net charge, which slightly increased in the presence of the tannin (Figure 2A). The slightly positive value can be attributed to the utilized hydration medium, i.e., PBS; however, the surface charge in all cases was practically zero and provided no electrostatic interactions between the particles.

The size of liposomes and DMPC–RT nanosystems was approximately 100–110 nm in all cases (Figure 2B). Analysis of the polydispersity index (PDI) (Figure 2C) allowed us to conclude that all liposomes (without and in the presence of RT) had rather homogenous size distribution.

As demonstrated in Figure 2B, both the pure liposomes and the hybrid DMPC–RT nanoparticles possessed roughly the same hydrodynamic diameter (approx. 100 nm). When comparing the PDI values, a range of 0.44–0.49 was noticed, indicating that all the formulated DMPC–RT complexes were mostly uniform with a homogenous size distribution (i.e., PDI = 0 stands for perfectly uniform liposomes, PDI = 1 stands for high polydispersity of the liposomes) [33].

In order to verify if the obtained liposomal–RT systems were stable over time (i.e., no aggregation observed), the measurements of the hydrodynamic diameter (D_h_) and PDI were additionally performed on days 1 and 5 after the day of preparation. The obtained results are presented in Table 1.

As observed in Table 1, pure liposomes (DMPC) and liposomes with the lowest concentration of RT (DMPC:RT1) are generally stable over time. Both the diameters and the PDI values are similar to day 0, after 1 and 5 days. A different effect was observed for liposomes with a 5 µM and 10 µM concentration of sumac tannin. For both systems, an increase in hydrodynamic diameter and PDI, in comparison to day 0, was observed. This shows that, in the presence of these concentrations of RT liposomes have a much lower stability and a tendency to form large aggregates with low homogeneity.

**Figure 3:**
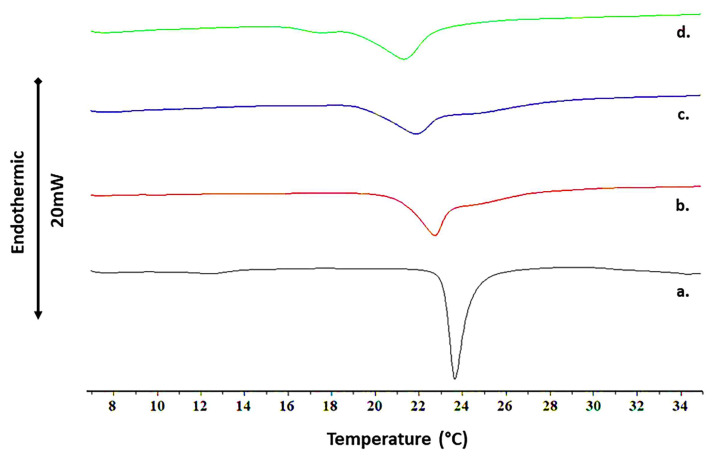
DSC heating profiles of a. DMPC, b. DMPC:RT1, c. DMPC:RT5, and d. DMPC:RT10 systems.

Polyphenols, including tannins, have the ability to change their thermodynamic parameters through interactions with membranes [26,32]. In order to define the influence of RT on the thermodynamic parameters of DMPC liposomes, DSC analysis was performed, and the results are presented below (Figure 3).

The pure DMPC phospholipids present one pretransition point between 12–13 °C (Please see line a in Figure 3) and a transition point at around 23.6 °C, typical for this lipid [34]. The presence of RT led to the disappearance of the pretransition of DMPC, which means that the RT molecules interact with the polar head groups and affect their mobility [35]. This is certainly associated with the H-bonding between the hydroxyl molecule groups and the lipid phosphate groups. This result is consistent with our previous findings regarding 1,2,3,4,6-penta-O-galloyl-β-D-glucose and 1,2-di-O-galloyl-4,6-valoneoyl-β-D-glucose [26]. RT led to a concentration-dependent decrease in the onset and peak temperatures of the DMPC main transition, as well as to the concentration-dependent change of the peak width that generally increased, except for the heating of DMPC:RT10. This indicates that the cooperativity of the system decreases as the amount of added tannin is increased, giving rise to new phases, probably due to raft/domain formation [36]. This is also caused by the inhomogeneous distribution and interaction of tannins onto the membrane.

Additionally, the existence of shoulders in all the lipid–tannin systems was observed as a result of the tannin effect that led to phase separation, especially in the highest RT amount (line d in Figure 3), where a new peak was almost formed at 17.5 °C. To better demonstrate the observed alterations of the thermal effects, the main thermodynamic parameters, i.e., transition enthalpy (ΔH_m_), temperature at which the thermal effect starts (T_onset,m_), gel to liquid–crystalline phase transition temperature (T_m_), and width of the transition at half-peak height (ΔT_1/2,m_), were calculated and are presented below (Table 2) [37].

The transition enthalpy (ΔH_m_) did not alter at all for any of the tannin concentrations. The decrease in enthalpy combined with the decrease in the transition temperature would mean fluidization of the system and less efficient lipid transition. However, in this case, the amount of energy required for transition was distributed in the various formed domains, which exist and transit in a wide temperature range [38]. As a result, we conclude that RT does not penetrate the inner part of the membranes when mixed with bilayers but rather interacts on their surface, promoting the observed thermodynamic alterations. To summarize, the interactions induced by the RT molecules lead to domain formation, with the resulting lipid domains absorbing and emitting cumulatively the same energy amount with the initial transition.

The appearance of shoulders occurs at higher temperatures than the main transition for the DMPC:RT1 and DMPC:RT5 bilayers but at lower temperature for DMPC:RT10. This indicates that there is a limit in tannin concentration, above which the interactions, adsorption, and domain formation on lipid bilayer is altered. This might reflect on the biological effect of the particular tannin molecules on biological membranes, where different domains are formed and relates to the thermodynamic equilibrium and metastability of the membrane. This transition resembles a “flip-flop” phenomenon in the minimum free energy of the system between the tannins and the lipid bilayer, where the molecules need to rearrange and interact differently with membranes in order to reach thermodynamic equilibrium, thus promoting the formation of a different in nature rafts/domains.

### 3.2. Influence on Lipid Order Parameter and Lipid Nanodomain Formation

**Figure 4:**
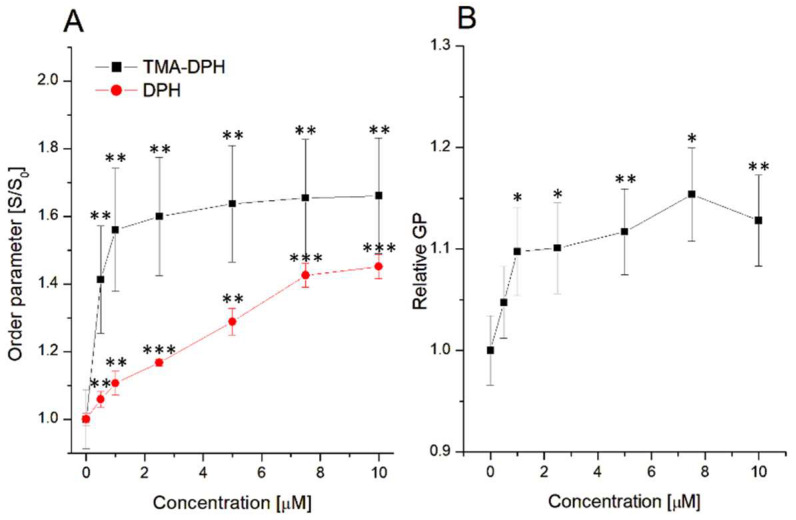
Changes of lipid order parameters in polar (◼) and hydrophobic (●) parts of liposomes (**A**), lipid domains formation (**B**). Statistical significance was estimated using paired t-test, with results compared to control (* *p* < 0.05; ** *p* < 0.001; *** *p* < 0.0001).

As described above, sumac tannin has a strong ability to change the thermal profile of DMPC phospholipids, leading to the promotion of lipid nanodomain (ND) formation. In order to confirm this assumption, studies of lipid order parameters and of ND formation in DMPC liposomes under the influence of RT were performed, and the results are presented below (Figure 4).

Based on the obtained results, it can be concluded that RT has the ability to change the lipid order parameters both in the hydrophilic as well as in the hydrophobic parts of DMPC liposomes. For both the polar and the nonpolar regions of liposomes, an increase in the order parameter was observed in comparison to the control system. This process is connected to the rising of the liposomal membrane rigidity. The same influence on the liposome’s rigidity was observed in our previous work, where PGG and 1,2-di-O-galloyl-4,6-valoneoyl-β-D-glucose (T1) interacted with DMPC liposomes [26]. On the other hand, the difference in the strength of the effect was noticed for the DMPC polar/nonpolar parts of the liposomes. These alterations are mostly connected with lipophilicity of polyphenols. For example, the rather nonpolar character of the PGG and T1 used evokes larger changes in the hydrophobic parts of the liposomes [26]. A similar relationship has been described for quercetin [32], which, as a hydrophobic compound, induced a larger decrease in fluidity at the nonpolar parts of the liposomes, as well as for curcumin [39], which, as a highly hydrophobic compound, induced a strong increase in lipid order parameters at the hydrophobic parts of the erythrocyte membranes. RT, as a more hydrophilic compound, triggered stronger changes in the polar regions of the liposomes (Figure 4A).

A strong influence of RT on the liposomal membrane (Figure 4A) as well as the changes in the temperature transition point, due to the presence of tannin (Figure 3), allows us to assume that formed lipid nanodomains are the result of sumac–liposome interactions. Therefore, fluorescence studies using a Laurdan fluorescent label were performed, and the results are demonstrated in Figure 4B. The increasing concentrations in sumac induced an increase in the generalized polarization (GP). This process is associated with the membrane fluidity and the lipid hydration [40,41] and corresponds to the increase in packing density of the polar regions of the liposomal membrane and domain formation, as DSC studies suggest, as well as having good correlation with TMA-DPH results. This is probably the consequence of the dehydration of DMPC polar heads. Similar results were observed for PGG interaction with liposomes [26].

### 3.3. Fluorescence Analysis of Sumac–Liposome Interactions

In order to better characterize the interaction between sumac and DMPC liposomes, based on the measurement of TMA-DPH fluorescence quenching, the biophysical parameters of the Stern–Volmer constant (K_SV_), quenching constants (k_q_), and binding constant (logK_b_) were calculated. TMA-DPH (marks polar parts of liposomes) has been used as a fluorescence donor since the RT interacted with the hydrophilic area of the studied vesicles.

Fluorescence quenching is most often described by the Stern–Volmer (SV) equation (Equation (1)) [42]:

(1) $$\frac{F_{0}}{F}=1+{\;K}_{\mathrm{SV}}\;\left[Q\right]$$

where F_0_ is the fluorescence without quencher,

F is the fluorescence in the presence of the quencher;
K_SV_ is the Stern–Volmer constant;
[Q] is the quencher concentration.

**Figure 5:**
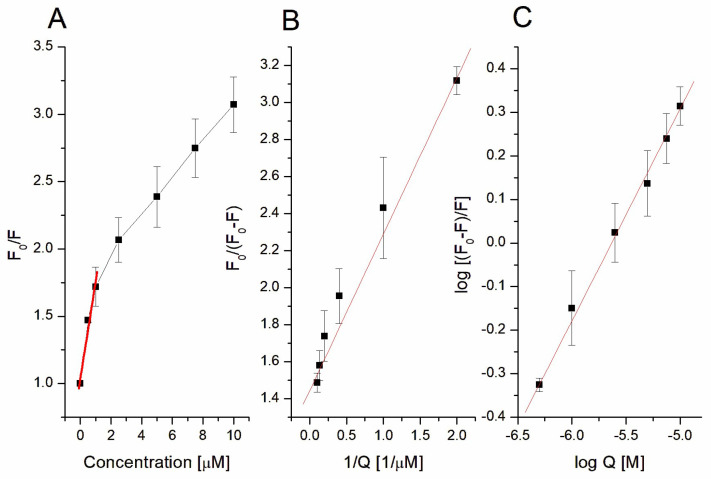
Stern–Volmer plot (**A**), modified Stern–Volmer plot (Lehrer plot) in normal (**B**) and double-logarithmic scale (**C**) of TMA-DPH fluorescent-stained DMPC liposomes.

Based on the above equation, the Stern–Volmer graph was plotted (Figure 5A). When high linearity of SV plot is observed, the single class of fluorophores is accessible for the quencher molecules and the two main quenching modes occur, i.e., static mechanism (with quencher–quenching molecule complex formation) or dynamic mechanism (based on collisional encounters between quencher and quenched molecules) [42]. Based on these studies, a high linearity was observed only for the first three points, giving a Stern–Volmer constant of K_SV_ = (7.65 ± 1.21) × 10^5^ M^−1^. Using this K_SV_ value, the quenching constant (k_q_), which allows us to obtain the information about the quenching mechanism, can be calculated based on the following equation (Equation (2)) [42]:

(2) $$k_{q}=\frac{K_{\mathrm{SV}}}{\tau_{0}}$$

where: k_q_ is the quenching constant,

K_SV_ is the Stern–ˆVolmer constant;
τ_0_ is the average lifetime of fluorophore molecules (5 × 10^−9^ s).

The quenching constant was calculated to be k_q_ = (1.53 ± 0.24) × 10^14^ M^−1^s^−1^, which is larger than the value of 2 × 10^10^ M^−1^s^−1^ and is the maximum scatter collision constant value. Thus, the interactions lead to the formation of complexes between RT and DMPC liposomes.

As mentioned above, the SV plot (Figure 5A) is not linear for the whole spectrum of RT concentration and present an up-down curvature towards the X-axis. This suggests the presence of two fluorophore populations with one of them not being accessible to the quencher [43]. In order to more holistically describe the sumac–liposome interaction, the modified Stern–Volmer equation (the so-called Lehrer equation, Equation (3)) was applied, leading to the regression line (red line) presented in Figure 5B.

(3) $$\frac{F_{0}}{F_{0}-F\;}=\frac{1}{f_{a}K_{\mathrm{SVa}}}\;\frac{1}{\left[Q\right]}+\frac{1}{f_{a}}$$

where

F_0_ is the fluorescence observed in absence of quencher;
F is the fluorescence observed in presence of quencher;
K_SVa_ is the effective Stern–Volmer constant for the accessible fluorophores;
*f_a_* is the fraction of accessible fluorophore.

According to Equation (3), the accessible Stern–Volmer constant was calculated to be K_SVa_ = (1.86 ± 0.22) × 10^6^ M^−1^, and the *f_a_* was 0.66 ± 0.05. When the fluorophore is completely accessible for the quencher, the *f_a_* value is equal or higher than 1 [44]. Since, in our studies, the fraction of accessible fluorophore is lower than 1 (i.e., 0.66 ± 0.05), it can be concluded that RT did not access all TMA-DPH molecules. The dissociation constant K_d_, as the inverse of *f_a_·K_SVa_*, was also calculated with Equation (3), with a value of K_d_ = (8.26 ± 0.56) × 10^−7^ M. Apart from the dissociation constants, the binding constant (logK_a_) was also calculated by using the double-logarithmic equation (Equation (4)) and with the resulting regression line being presented in Figure 5C.

(4) $$\log_{10}\;\frac{F_{0}-F}{F}={n\;\log}_{10}\;\left[Q\right]+{\;\log}_{10}K_{a}$$

where

F_0_ is the fluorescence observed in absence of quencher molecules;
F is the fluorescence observed in presence of quencher molecules;
K_a_ is the binding constant;
Q is the quencher concentration.

Based on Equation (4), the logK_a_ was calculated at logK_a_ = 2.66 ± 0.26. Based on the calculated dissociation (K_d_) and association (logK_a_) constants, it can be concluded that RT shows high affinity for interaction with DMPC liposomes. The results are in good agreement with the above data obtained from DSC and correspond well with the changes of the lipid order parameter, indicating that RT interacts with the surface of liposomes. A similar, strong interaction with DMPC liposomes was observed during our previous work with PGG and T1 [26]. Earlier, Reis et al. had already demonstrated that PGG as well as EGCG (epigallocatechin gallate) interact with large, unilamellar di-stearoyl-glycerophosphatidylcholine:cholesterol (DSPC:Chol) liposomes [31]. However, the K_d_ and the logK_a_ values for PGG calculated by Reis et al. were somewhat different compared to the ones for RT. These discrepancies may be the result of different liposome composition (DSPC:Chol vs. DMPC), fluorescent labels (2-AS vs. TMA-DPH), or the structure of molecules (PGG has 5 gallic acid residues whereas RT carry 7 gallic residues) between the different studies.

### 3.4. Antibacterial Activity of Sumac–Liposome Nanocomplexes

The aforementioned results clearly prove that RT strongly interacts with DMPC liposomes, leading to the formation of an RT–liposome hybrid nanosystem. It is well known that liposomes can be widely used as nanocarriers of drugs [45,46], as well as different types of natural plant compounds, e.g., resveratrol, quercetin, fisetin, sylimarin [47], ulvan (polysaccharide from green seaweeds) [48], and curcumin [39]. Tannins are plant polyphenols that present strong antibacterial activity [49,50]. The RT investigated during our work also demonstrates antistaphylococcal activity, as described previously [8]. In order to verify the effect of the encapsulated liposome RT against bacteria, the hybrid sumac–liposome nanoparticles were formulated, and their antibacterial activity was analyzed by measuring the minimum inhibition concentration (MIC). The results are presented in Table 3.

As observed in Table 3, the formulated hybrid RT–DMPC nanoparticles have much stronger antibacterial activity against Gram-positive bacteria, especially *S. aureus* 8325-4 and *S. epidermitis* ATCC 14990 (8 times lower MIC in comparison with RT alone). A weaker activity was noticed against *S. aureus* ATCC 700699 and *B. cereus* ATCC 13061, with the antibacterial effect being, however, still higher than that of pure sumac (4 times and 2 times, respectively). These observations suggest that such complexation of RT with liposomes increases its antimicrobial activity. Similar results were obtained in the recently published work of our team with curcumin, where the complexation with lipid–polymer liposomes increased the antibacterial activity of curcumin against *S. aureus* NCTC 5655 [39]. The increase in activity after complexation with the liposomes was also demonstrated by the Risaliti team regarding liposomes loaded with *Salvia triloba* and *Rosmarinus officinalis* essential oils [51]. The liposome complexes demonstrated a higher antibacterial activity against Gram-negative *Klebsiella pneumoniae* in comparison to unformulated *Salvia* and *Rosmarinus* oils [51]. However, contrary to the findings of Risaliti et al., our hybrid RT–liposome nanoparticles, compared with RT, had much lower antibacterial activity in relation to Gram-negative bacteria, i.e., *E. coli* ATCC 35218 and *P. aeruginosa* ATCC BAA-1744. These differences may be attributed to discrepancies in the strains of bacteria used, in phospholipids used, in liposome preparations, and in the type of active compounds examined.

Based on the above results, it may be concluded that the formulation of hybrid RT–liposome nanoparticles allowed us to obtain a new biomaterial which shows high antibacterial activity against Gram-positive strains.

## 4. Conclusions

This present work focused on the study of the interactions between 3,6-bis-O-di-O-galloyl-1,2,4-tri-O-galloyl-β-D-glucose (RT, sumac tannin) and DMPC liposomes as well as on the formulation and evaluation of the antimicrobial activity of a new, hybrid RT–liposome biomaterial. According to obtained data, it can be concluded that RT has a strong affinity to liposomes and interacts with them, leading to the formation of RT–liposome complexes. This leads to alteration of the thermodynamic properties of DMPC-liposomes and an increase in the lipid order parameter, which is associated with an increase in the liposomal membrane rigidity and formation of lipid nanodomains. The RT–liposome hybrid nanobiomaterials demonstrated much stronger antibacterial activity against Gram-positive bacteria, such as *S. aureus*, *S. epidermitis* and *B. cereus,* in comparison to RT alone. On the other hand, changes in size and the polydispersity index of liposomes in the presence of RT over time allowed us to conclude that the complexes do not exhibit high colloidal stability. In conclusion, we proved that using the high affinity of RT to liposomes, new, functional nanobiomaterials with strong antibacterial activity and potential pharmacological applications can be formulated, but further research is still required to optimize these formulations and establish them as therapeutic products.

## Figures and Tables

**Table 1:** Hydrodynamic diameters and polydispersity index (PDI) values of DMPC and DMPC–RT liposome systems over time.

t (Days)	DMPC		DMPC:RT1		DMPC:RT5		DMPC:RT10	
	D_h_ (nm)	PDI	D_h_ (nm)	PDI	D_h_ (nm)	PDI	D_h_ (nm)	PDI
0	113.5 ± 0.5	0.453 ± 0.009	113.6 ± 1.0	0.441 ± 0.006	97.5 ± 2.7	0.492 ± 0.005	107.2 ± 1.6	0.466 ± 0.009
1	131.5 ± 2.3	0.568 ± 0.012	132.6 ± 1.9	0.512 ± 0.006	389.9 ± 10.0	0.742 ± 0.233	215.9 ± 4.1	0.703 ± 0.096
5	146.9 ± 2.0	0.646 ± 0.012	143.0 ± 2.4	0.515 ± 0.018	203.2 ± 3.1	0.623 ± 0.008	237.5 ± 2.2	0.680 ± 0.120

**Table 2:** Calorimetric profiles of DΜPC:RT bilayers in PBS (pH = 7.4) after heating.

Sample	RT [µM]	T_onset,m_ (°C)	T_m_ (°C)	ΔT_1/2,m_ (°C)	ΔH_m_ (kJ/mol)	T_onset,s_ (°C)	T_s_ (°C)	ΔT_1/2,s_ (°C)	ΔH_s_ (kJ/mol)
DMPC	-	22.94	23.37	0.77	29.47	10.84	12.32	1.62	1.33
DMPC:RT1	1	21.00	22.61	1.50	29.41	-	-	-	-
DMPC:RT5	5	19.26	21.75	2.41	29.54	-	-	-	-
DMPC:RT10	10	18.60	21.16	2.35	29.29	-	-	-	-

**Table 3:** Antibacterial activity of RT and hybrid nanocomplex (RT–liposome complex) demonstrated as MIC values (μM; concentration in relation to sumac tannin).

	*S. aureus* ATCC 700699	*S. aureus* 8325-4	*S. epidermitis* ATCC 14990	*B. cereus* ATCC 13061	*E. coli* ATCC 35218	*P. aeruginosa* ATCC BAA-1744
	MIC [μM]					
RT	62.5	15.625	62.5	62.5	250	250
RT-DMPC nanoparticles	15.625	1.95	7.81	31.25	>500	>500

## Data Availability

Not applicable.
